# Learning experiences of final-year student midwives in labor wards: A qualitative exploratory study

**DOI:** 10.18332/ejm/111802

**Published:** 2019-08-29

**Authors:** Joeri Vermeulen, Wim Peersman, Matthias Waegemans, Gerlinde De Clercq, Leonardo Gucciardo, Monika Laubach, Eva Swinnen, Katrien Beeckman, Ronald Buyl, Maaike Fobelets

**Affiliations:** 1 Department Health Care, Knowledge Centre Brussels Integrated Care, Erasmus University College Brussels, Brussels, Belgium; 2 Social and Community Work, Odisee University College, Brussels, Belgium; 3 Faculty of Medicine and Health Sciences, Department of Rehabilitation Sciences, Ghent University, Ghent, Belgium; 4 Faculty of Medicine and Pharmacy, Vrije Universiteit Brussel (VUB), Brussels, Belgium; 5 Department of Obstetrics and Prenatal Medicine, University Hospital Brussels, Brussels, Belgium; 6 Faculty of Physical Education and Physiotherapy, Rehabilitation Research, Vrije Universiteit Brussel (VUB), Brussels, Belgium; 7 Faculty of Medicine and Pharmacy, Department Medical Sociology, Vrije Universiteit Brussel (VUB), Brussels, Belgium; 8 Nursing and Midwifery Research Unit, University Hospital Brussels, Brussels, Belgium; 9 Faculty of Medicine and Health Sciences, Centre for Research and Innovation in Care (CRIC), Midwifery Research Education and Policymaking (MIDREP), University of Antwerp, Antwerp, Belgium; 10 Faculty of Medicine and Pharmacy, Department of Public Health, Biostatistics and Medical Informatics Research Group, Vrije Universiteit Brussel (VUB), Brussels, Belgium

**Keywords:** midwifery education, midwifery students, clinical placements, labor wards, student experiences

## Abstract

**INTRODUCTION:**

Clinical placements are an integral part of midwifery education and are crucial for achieving professional competencies. Although students’ experiences on placements have been shown to play a meaningful role in their learning, they have received scant attention in the literature. The aim of this paper is to describe the learning experiences of final-year student midwives in labor wards within the Brussels metropolitan region, Belgium.

**METHODS:**

A qualitative exploratory study was conducted using two focus groups (N=20). Data analysis included: transcription of audio recordings, thematic content analysis with coding into recurrent and common themes, and broader categories. Discussions among researchers were incorporated in all phases of the analysis for integrity and data fit.

**RESULTS:**

Data analysis identified the following categories as determining student learning experiences in labor wards: 1) managing opportunities, 2) being supported, and 3) dealing with the environment. Overall, respondents were positive and enthusiastic about their learning experiences, although some felt tense and unprepared. Students expressed concerns about differences in learning opportunities between placements and found it challenging to achieve all competencies. Student learning experiences were enhanced when they had placements for longer periods with the same supportive mentors.

**CONCLUSIONS:**

Factors related to students’ functioning, the healthcare professional, midwifery education and hospital environment affected their learning in labor wards. The combination of a more persevered preparation of students and mentors, together with a student-centered organization of placements, is crucial to create a positive learning experience for midwifery students in labor wards.

## INTRODUCTION

Midwifery education is the foundation for high-quality, safe and evidence-based care for women and their new-borns^1^. An integral part of midwifery education programs are clinical placements, which are crucial for achieving the required professional competencies^2,3^. The impact of placements cannot be underestimated, as they may either encourage or impede a positive learning experience for student midwives^4,5^. Especially, the labor ward is a challenging environment that offers a wide range of authentic learning opportunities^6^. Labor wards are rather unstructured environments, inherently offering diverse and inconsistent learning opportunities^7^. As placements in labor wards are indispensable for the development of professional competencies, students need to be adequately supported and prepared for them.

A successful teaching strategy involves learning experiences in labor wards. Although learning experiences have been shown to play a meaningful role in students’ acquisition of professional competencies in labor and birth care, they have received scant attention in the literature^8^. Previous studies indicated that student midwives find it difficult to gain access to learning experiences in labor wards, because they are first expected to prove themselves to be accepted by the midwives^6,9^. Other studies pointed out the pressure to ‘chase’ as many births as possible during the final placement to meet the required criteria for professional competencies in midwifery^7,9,10^. Others reported on the perceived competition between student midwives and medical students for learning experiences^7,11^.

As there is a knowledge gap about strategies to adequately prepare students for the reality of practice^12^, it would be helpful to examine how these learning experiences in the labor ward can be enhanced. The challenge here is to offer high-quality placements with access to learning experiences, where students will be instructed by knowledgeable, devoted mentors^13^.

In Belgium, all student midwives are expected to acquire professional competencies in all fields of midwifery^14^, according to national and European legislation^15^ as well as the International Confederation of Midwives’ Global Standards for Midwifery Education^16^. This is a challenge in the current maternity care context, as placements are mainly set in hospitals where most births are performed by obstetricians. Consequently, it is difficult for Belgian students to obtain the necessary professional competencies in labor and birth care. In this context, it is crucial that students are well prepared for their placement and can acquire strategies to support their learning in labor wards. The aim of the present study is therefore to explore the learning experiences of final-year student midwives in labor wards within the Brussels metropolitan region, Belgium.

## METHODS

### Design

To capture the learning experiences of final-year student midwives in labor wards, a qualitative exploratory study was organized with interviews of two focus groups.

### Setting

The study was performed in 2018 at Erasmus University College Brussels in Brussels, Belgium, with 210 student midwives enrolled in the 3-year direct-entry program, including 46 final-year students. During the program, students spend approximately 50% of their study time in clinical practice (38 weeks, including minimum 20 weeks in labor wards). Students have scheduled placements in both low-risk and high-risk settings, in public and private healthcare settings principally within the Brussels metropolitan region. Each student is assigned a supervisor, from the university college, who coaches and supports them during the placement. Typically, midwives from the placement are assigned as mentors and, together with the supervisor, provide practical education, give feedback and assess the student.

### Data collection

Students were informed about this study and were invited by email to participate four weeks before the planned interviews. A reminder was sent two weeks prior to the interviews. Interviews were planned at university college, neither the interviewer (MF, female) nor the observer (MW, male) was involved in teaching or assessments in the program, to avoid any bias. Informed consent, permission for audio recording and sociodemographic data and information about previous clinical placements (i.e. duration, hospital, municipality) were collected from participants.

Semi-structured interviews were held in Dutch. An interview guide with open-ended questions, derived from a review of the literature^6,9,11^, was used (Table 1).

**Table 1:** Interview guide

**Engagement question**
Can you describe your overall experiences of your clinical placements in labor wards?
**Exploring questions**
Which were the most important learning experiences during your clinical placements in labor wards?
Which factors facilitated your learning experiences during your clinical placements in labor wards?
Which factors hindered your learning experiences during your clinical placements in labor wards?
Could you acquire the requested competencies during your clinical placements in labor wards?
Which competencies were difficult to achieve during your clinical placements in labor wards?
Are there issues that would have improved your learning experiences during your clinical placements in labor wards?
**Exit questions**
Is there anything additional you would like to say about your learning experiences during your clinical placements in labor wards?
Of all things discussed today, which do you think is the most important?
**Probes in order to minimize misunderstandings**
Can you please tell more about this?
Help me understand what do you mean by that?
Can you give an example of that?
Can you tell me something else about that?

During the focus groups, the interviewer asked questions and facilitated the discussion, while the observer digitally recorded the interview. All audio recordings were securely stored in an on-site locked facility, which was only accessible to the researchers. No data were shared or discussed with other colleagues, and to maintain anonymity all identifying information was removed. Ethical approval was obtained from the University Hospital Brussels and the Vrije Universiteit Brussel (VUB), Belgium, in November 2017 (registration number: B.U.N./143/201/733/426).

### Data analysis

The audio recordings were transcribed by JV, who did not participate in the interviews. Both the interviewer and the observer reviewed the transcriptions in the weeks following the interviews. A thematic content analysis formed the basis of the analysis; the whole data set was coded into recurrent and common themes. The coding was independently performed by two coders (JV, WP), who first familiarized themselves with the content of the transcripts before individually coding sections into meaning units (i.e. themes), in line with the study aim^17^. Next, the coders, the interviewer and the observer discussed themes and created broader categories to optimally reflect the data. This involved a rigorous comparison of the transcripts, on paper and by means of NVivo version 11 (QSR International). One coder (JV) organized the results and both coders and the interviewer checked the final analysis for integrity and fit with the data, and significant examples of each theme were selected and translated into English.

## RESULTS

Both focus groups lasted approximately 40 minutes, of the 46 invited final-year students, 20 (43.5%) participated in the interviews, all aged 20–37 years (mean: 22.6, SD: 4.0 years) (Table 2).

**Table 2:** Characteristics of participants

*Focus group*	*Number of participants*	*Age (Years)*	*Clinical placements in labor wards within the Brussels metropolitan region (Weeks per student)*	*Clinical placements in labor wards outside the Brussels metropolitan region (Weeks per student)*
	*N*	*Median (Range)*	*Median (Range)*	*Median (Range)*
1	11	22.0 (20–28)	9.0 (0–17)	0 (0–10)
2	9	21.0 (20–37)	12.0 (4–25)	0 (0–6)
Total	20	21.5 (20–37)	10.5 (0–25)	0 (0–10)

Student learning experiences in labor wards were clustered into three categories: 1) managing opportunities, 2) being supported, and 3) dealing with the environment. Each category embraced three themes (Table 3), discussed below. The implication from these categories and themes is that the positive operationalization of the theme, e.g. taking initiative, results in positive learning opportunities whereas not taking initiative may inhibit or prevent learning experiences.

**Table 3:** Categories and themes determining student learning experiences in labor wards

*Categories*	*Themes*
Managing opportunities	Taking initiative
	Dealing with emotional uncertainty
	Seeking experiences and clinical opportunities
Being supported	Developing professional relationships
	Experiencing reflective mentorship
	Trusting supervisors
Dealing with the environment	Preparing for the reality of clinical practice
	Understanding contexts and recognizing opportunities
	Being successful in the competitive environment

### Managing opportunities

In general, students described clinical placements as positive, motivating and instructive. They reported that the access to learning experiences clearly depended on their own ability to manage opportunities to take the initiative, dealing with uncertainty and seeking learning opportunities.

#### Taking initiative

If students could take the initiative, they found this a very important condition to gain access to learning experiences that enhanced their learning.

*‘To follow up on a woman’s labor completely … with the midwife’s support, but above all to be able to do it alone, and also get the chance to take the initiative, … so I learned a lot’*. (FG1)

Self-starters were more likely to get a variety of learning opportunities, which contributed to their professional growth if they could make optimal use of them.

#### Dealing with emotional uncertainty

The labor ward was experienced as a challenging environment with numerous learning opportunities. Students felt that the mentors and supervisors often expected their performance to perfectly match with what they had learned in education. Consequently, some students were tense and uncertain, felt pressured to prove themselves and were unsure if they could meet the requirements.

*‘You don’t know what to expect, … so before you start [the placement] you are wondering “will I be able to, … or not?” and that contributes to stress and uncertainty, so asking yourself “am I good enough?” …’* (FG2)

If these students felt tense and uncertain, they avoided taking the initiative to facilitate their learning. In some cases, this negatively affected student relationships with the staff and hindered their access to learning experiences. Although students believed that they learned much from working in a real-life setting and observing the midwife’s work, some competencies were reported to be more difficult to acquire than others.

#### Seeking experiences and clinical opportunities

Our respondents were mostly excluded from learning opportunities in acute situations. They were usually not allowed to actively participate and felt forced into an observer role.

*‘In case of pathologies and such, for example a postpartum bleeding or something like that, the student is like a bit … pushed aside …., I regret that, because then you cannot learn like …, you are able to observe but you are not dealing with stuff that contributes to real experiences …’* (FG2)

When the students were confronted with an acute situation, they had little opportunity to reflect, as the mentors usually did not debrief them or only in a very perfunctory manner. In their seeking for clinical opportunities, students reported that reflection and analysis after an acute situation would contribute to their learning and improve their experiences.

Some students found it difficult to acquire basic, essential competencies, which was often a source of stress and anxiety. Access to learning experiences in vaginal examination, episiotomy and perineal repair was limited. Some mentors reportedly did not consider episiotomy and suturing that are essential midwifery competencies. Vaginal examinations were particularly hard to practise upon admission and if the woman chose to forego an epidural analgesia. Additionally, it was hard to develop any competence in writing reports and briefing other team members and health professionals.

*‘I often asked [the mentors] if I could assist in filling in the file or observe them doing that, because we need to do that eventually, now at the end of the year, … but they always ignore that, “yes but, the room needs to be cleaned up …” [laughter and endorsement from other students] …. Yes, they always send you away, so you don’t get a chance to … [learn that competency]’*. (FG1)

### Being supported

Students’ learning was facilitated if they felt supported, which was shown to be highly dependent on the development of professional relationships with the mentor, if the mentor was reflective and when the student trusted the supervisor.

#### Developing professional relationships

The development of professional relationships appeared to be complex. The busy and stressful environment sometimes made it difficult to establish professional relationships, as did the fact that placements are organized in different hospitals and are often rather short. A professional relationship with the mentor was identified as being crucial to the students’ learning experiences, as it increases their chances of being accepted as part of the team and enhances access to learning experiences. In most cases, a professional relationship was established between the student and mentor. In other cases this proved to be difficult, which affected student learning experiences.

*‘The [mentor’s] personal opinion might not always be positive, and they show it, …. in my case, I am somewhat older, and some [mentors] found me too old to do that [studying midwifery]. They were very explicit in their opinion, and then it is difficult to [cynical laughter] still have a meaningful day after you heard that’.* (FG2)

Occasionally a sense of distance was reported, e.g. when the mentor did not know the student’s name or what year she was in, even after they had been working closely together during a long shift. Furthermore, it was challenging to connect with mentors when their expectations were unrealistic.

#### Experiencing reflective mentorship

Midwives were categorized as either supportive or unsupportive towards students’ learning. Students developed their competencies if the mentors supported them and were willing to let them perform. Respondents reported that they had the best learning experiences if the mentor was supportive and conscious of their learning needs. Supportive mentors fulfilled a constructive and reflective role. They challenged the students in taking initiatives to gradually make independent decisions, thus providing instructive learning opportunities.

*‘I had to reflect, “it has been a while since we examined the woman, should we go see her again?” That is what I like, that reflection. It is not like “now we are going to do that” …’* (FG1)

In contrast, unsupportive midwives were described as dictating and unreflective, which limited student access to learning experiences. Students felt that these mentors did not take them seriously and preferred them to perform supportive tasks only, and did not challenge them to acquire professional competencies.

Respondents stressed the need for more continuity of mentorship during placements. This contributes to trust, creates a connection and increases the likelihood to develop a professional relationship. Continuity of mentorship was seen as improving the monitoring and progress of their learning and being assigned to the same mentor was regarded as beneficial for their learning.

*‘I did a placement in X [name hospital] and there I worked with the same midwife the whole time, always the same shifts … you are monitored so much better, because she knew what I had done the day before, … because she knew what I was capable of. That is really great, to have someone who follows up on you’*. (FG1)

#### Trusting supervisors

Supervision from the university college was felt as being beneficial to student learning at clinical placements; supervisors took on an advocacy role that supported students in their learning. Supervisors facilitated access to learning experiences, particularly if they collaborated closely with the mentors. A prior professional relationship between the supervisor and the mentor was perceived to be advantageous to the learning experience, as the supervisor could more easily put things into perspective.

*‘Sometimes it is easier if the supervisor had also worked in those hospitals, because then they know ‘oh, yes that midwife* … *I know her, she* was *my colleague and* was *always going on about that …’, so then I know what I should pay attention to, because if the supervisors say so …’* (FG1)

However, some students found the supervisor’s role to be ambivalent, especially when they had worked at the placement or were friends with some of the mentors. This affected the extent to which students trusted their supervisor, as they did not know where the allegiances of the supervisor lay.

### Dealing with the environment

According to the students, their learning was strongly influenced by the extent to which they felt prepared for reality of practice, could understand different contexts and recognize learning opportunities and could deal with the competitive environment.

#### Preparing for the reality of clinical practice

Students identified adequate preparation for placement as crucial in their effective learning, when adequately prepared they were more confident and scouted more actively for learning experiences. Some students felt especially unprepared at the beginning of their second year of studies. Students who had a limited knowledge of pathology were often not taken seriously by the mentors and were more likely to be excluded from learning experiences. Respondents who did not feel adequately prepared for practice were challenged to meet the mentor’s expectations. In some cases, students found these expectations to be unrealistically high, which negatively affected their self-confidence and hindered their further learning experiences.

*‘I did my first placement at X [name hospital], where you get a lot of pathologies, … and they expected me to know about things that I had never, … that affected my self-confidence, and … the rest of the placement, …. I just think they had the wrong expectations’.* (FG2)

Some students were unprepared for the tension between the perceptions of midwifery they had and the actual midwifery practice that they observed. Students defined ‘ideal practice’ as practice preserving physiology, which is evidence-based and in line with what they had learned. In contrast, actual practice was referred to as high-risk, with many interventions that were not always evidence-based, and considerably more complex than what they had been prepared for.

Respondents emphasized that the education program should be more focused on the actual practice by using authentic learning materials to improve their readiness for practice and avoid a reality shock.

*‘The idea was to perform physiological births, but I have to admit that I did not see that a lot, …. Probably the footage, … more real or another preparation, … before placement, so that you are better prepared. It is not like the romantic world that we were shown, baby clean and pink …’* (FG1)

Students described remarkable differences in hospital policies and organizational cultures. They noticed a significant variation in the number of learning experiences and the practices and approaches used by health professionals.

#### Understanding contexts and recognizing opportunities

Midwives’ working styles differed across the different hospitals, and incongruences with what students had been taught often led to confusion. Some practices were favored in one hospital and discouraged or criticized in another.

*‘And if you did placements in different hospitals, then you notice differences in practices, that on some occasions you do something in a particular hospital but if you do it the same way in another hospital that they, … blame you, that it is not acceptable for them’*. (FG2)

Students felt that they first needed time to adjust to the working style of the mentor, before getting access to learning experiences.

Respondents observed different workloads in different hospitals. In most hospitals, labor wards were experienced as busy and stressful. Although busy placements were perceived as providing more access to learning experiences, some respondents felt uncomfortable with the difficulties that these placements posed. At the same time, students did not always favor calmer labor wards either, as these offered a lower number of learning opportunities. Nevertheless, calmer wards were found to give students a better overview, which was comforting and enhanced the quality of learning.

*‘I did a placement in X [name hospital], and that was my favorite placement ever, … yes I was much quieter than …., yes I did placements in Y and Z [names of two busy hospitals], yes it was much quieter than in those two hospitals, but I liked it because you could follow up on one patient in a decent way, and really, in all tranquility … Because in Z you then have three patients or something and it is like you don’t have a clear overview anymore’.* (FG2)

#### Being successful in the competitive environment

It was emphasized that other medical or midwifery students were often present in calm labor wards, which created a competitive atmosphere. Students felt that they had to fight to be successful and get any learning experiences.

*‘I did my practice in X [name hospital], … the learning opportunities were limited there, because you barely have patients. It is super quiet, and … you were often confronted with a lot of other students, so if there* was *a patient …, then you had to fight with that student in order to have a patient’.* (FG2)

If there were fewer students on the ward, the quality of their learning experiences was enhanced and there was time to build a trusting relationship between the health professionals, which in turn facilitated access to learning experiences.

## DISCUSSION

This study provided information about the learning experiences of final-year student midwives in labor wards within the Brussels metropolitan region, Belgium. Data analysis identified the following categories as determining student learning experiences in labor wards: 1) managing opportunities, 2) being supported, and 3) dealing with the environment.

Overall, the respondents were positive and enthusiastic about their experiences. Nevertheless, they also revealed multi-focal challenges in facilitating their access to learning experiences. Students’ learning experiences were optimized when they found themselves in a safe, supportive environment^8^ where they could take initiative. The mentoring midwives, specifically, were found to be the gatekeepers of students’ access to learning experiences. We found that the development of a professional student-mentor relationship is complex and, as documented, the strength of that relation is the most determining factor in students’ learning process^18^.

The effectiveness of mentors’ investment in students’ learning becomes apparent after a longer placement period, which encourages students and mentors to develop a professional relationship and facilitates access to learning experiences. Students highlighted the importance of the continuity of mentorship and placement to become familiar with the environment and culture. In fact, our results confirm previous findings that a lack of continuity of mentorship hinders students’ access to learning experiences^8^. Furthermore, continuity gave them the opportunity to consolidate learning and establish positive relationships with their mentors. It is therefore advisable to couple students to one or a small group of supportive mentors, carefully select this group and prepare them for this role^19^ using an evidence-based mentorship program^20,21^. This raises the matter of adequate staffing and sufficient numbers of mentors^22^, and highlights the problems faced when one midwife has to meet the demands of dual roles of both midwife and mentor^23^.

Despite their preparation, some students felt tense and uncertain prior to their placement. They mainly attributed this to not feeling prepared for acute situations; however, many of our student midwives experienced such situations early in their placements. As our respondents emphasized, the need for more authentic learning material, new technologies such as virtual reality and serious gaming warrant further consideration^24^. It should be further explored how these advancements may contribute to preparing students for placements and developing competencies that are now difficult to achieve (e.g. vaginal examinations, perineal suturing).

Students recounted that what they had been taught was not necessarily what they observed in practice. The focus on physiological birth in the early stages of the curriculum may not sufficiently reflect actual practice. Some students were confused by those inconsistencies, which is confirmed in the literature^10,25^. Events responsible for the lack of congruence between actual midwifery practice and ideal perceptions of midwifery practice could be addressed through role play and peer reflection^26^. As peer reflection is recognized as a powerful learning strategy in midwifery education^27^, we recommend that educators work together with mentors and students to analyze incongruences and make them debatable. Increased digital learning, a more extensive use of simulation learning and more simulation sessions may be helpful to prepare students for the reality of clinical practice^28^, although this remains challenging particularly when the medical model is dominant^29^.

Previous studies, exploring midwifery students’ readiness for practice, have shown that midwifery students lack competency in areas where they are supposed to practice independently^6,9,30^. Finally, as active participation in acute situations and immediate debriefing were considered an important learning experience, we suggest structurally embedding multidisciplinary debriefings at placements in these contexts. Our students expressed concern about the diverse size of clinical placements and differences in learning opportunities, which is in line with international findings^7,11^. Over the last years, there has been an increase in the number of medical and midwifery students at placements, which has created a competitive atmosphere in terms of learning opportunities.

### Strength and limitations

The strength of our study is that it has the potential to inform educators, mentors and hospital managers to implement appropriate support strategies to optimize student learning. Some limitations of this study need to be addressed. Our data only represents the opinions of students from one setting, which reduced the variety of responses. A more diverse sample, with students from other regions, might have produced different insights. However, final-year students have already done more placements in labor wards and may have experienced more diverse learning situations, which actually enriches data collection. Since participants graduated one month before data analysis was completed (July 2018), neither the transcripts nor our findings were discussed with them. We improved the reliability of our results by using investigator triangulation and including both supportive and dissenting literature in the discussion section. More research is warranted to better understand the connection between students’ experiences and learning. Further research is required to identify the needs and concerns of the mentors and supervisors^23^. Finally, future studies should focus on strategies to optimize student learning experiences in labor wards.

## CONCLUSIONS

This study contributes to our understanding of how student midwives experience learning opportunities in labor wards. Although our participants were positive and enthusiastic about their learning experiences, they also identified a range of factors that affected their learning experiences. These factors were related to their functioning as well as to the healthcare professional, midwifery education and the hospital. The combination of a more persevered preparation of students and mentors by the university college together with student-centered organization of clinical placements, by placing fewer students at placements for longer periods with a limited number of supportive mentors, is crucial to facilitate a positive learning experience for midwifery students at labor wards.
