# Supplementary material for: Learning experiences of final-year student midwives in labor wards: A qualitative exploratory study
Source: Eur J Midwifery. 2019 Aug 29;3:15. doi: 10.18332/ejm/111802 (PMC7839143; doi:10.18332/ejm/111802)
Supplement: Supplementary file 1 [file EJM-3-15-s1.pdf]

No. Item	Guide questions/description	Reported in Section
**Domain 1: Research team and reflexivity**		
Personal Characteristics		
1. Interviewer/facilitator	Which author/s conducted the interview or focus group?	Methods/data collection
2. Credentials	What were the researcher's credentials?	Methods/participants and data collection
3. Occupation	What was their occupation at the time of the study?	Title page
4. Gender	Was the researcher male or female?	Methods/data collection
5. Experience and training	What experience or training did the researcher have?	Title page<br><br>The senior researcher/last author (interviewer and supervisor PhD), the second author (supervisor, PhD), and 4 co-authors (co-readers with PhD) have an extensive experience in both quantitative and qualitative studies.
Relationship with participants		
6. Relationship established	Was a relationship established prior to study commencement?	Methods/data collection
7. Participant knowledge of the interviewer	What did the participants know about the researcher? e.g. personal goals, reasons for doing the research	Methods/data collection and consent form
8. Interviewer characteristics	What characteristics were reported about the interviewer/facilitator? e.g. Bias, assumptions, reasons and interests in the research topic	Methods/data collection and consent form
**Domain 2: study design**		
Theoretical framework		
9. Methodological orientation and Theory	What methodological orientation was stated to underpin the study? e.g. grounded theory, discourse analysis, ethnography, phenomenology, content analysis	Methods/data analysis
Participant selection		
10. Sampling	How were participants selected? e.g. purposive, convenience, consecutive, snowball	Methods/participants
11. Method of approach	How were participants approached? e.g. face-to-face, telephone, mail, email	Methods/participants
12. Sample size	How many participants were in the study?	Results and table 2
13. Non-participation	How many people refused to participate or dropped out? Reasons?	Discussion
Setting		
14. Setting of data collection	Where was the data collected? e.g. home, clinic, workplace	Methods/participants
15. Presence of non-participants	Was anyone else present besides the participants and researchers?	Methods/data collection
16. Description of sample	What are the important characteristics of the sample? e.g. demographic data, date	Methods and results
Data collection		
17. Interview guide	Were questions, prompts, guides provided by the authors? Was it pilot	Methods/data collection

	tested?	
18. Repeat interviews	Were repeat interviews carried out? If yes, how many?	Methods/data collection
19. Audio/visual recording	Did the research use audio or visual recording to collect the data?	Methods/data collection
20. Field notes	Were field notes made during and/or after the interview or focus group?	Methods/data collection
21. Duration	What was the duration of the interviews or focus group?	Results
22. Data saturation	Was data saturation discussed?	Methods/data analysis
23. Transcripts returned	Were transcripts returned to participants for comment and/or correction?	Discussion
**Domain 3: analysis and findings**		
Data analysis		
24. Number of data coders	How many data coders coded the data?	Methods/data analysis
25. Description of the coding tree	Did authors provide a description of the coding tree?	Table 3: Categories and themes
26. Derivation of themes	Were themes identified in advance or derived from the data?	Methods/data analysis
27. Software	What software, if applicable, was used to manage the data?	Methods/data analysis
28. Participant checking	Did participants provide feedback on the findings?	Discussion
Reporting		
29. Quotations presented	Were participant quotations presented to illustrate the themes/findings?<br>Was each quotation identified? e.g. participant number	Results
30. Data and findings consistent	Was there consistency between the data presented and the findings?	Results
31. Clarity of major themes	Were major themes clearly presented in the findings?	Results/discussion/conclusion and table 3: Categories and themes
32. Clarity of minor themes	Is there a description of diverse cases or discussion of minor themes?	Results

Supplementary Table: Consolidated criteria for reporting qualitative studies (COREQ): 32-item checklist

© 2019 Vermeulen J.
